# Genome-Wide Association Analyses in 128,266 Individuals Identifies New Morningness and Sleep Duration Loci

**DOI:** 10.1371/journal.pgen.1006125

**Published:** 2016-08-05

**Authors:** Samuel E. Jones, Jessica Tyrrell, Andrew R. Wood, Robin N. Beaumont, Katherine S. Ruth, Marcus A. Tuke, Hanieh Yaghootkar, Youna Hu, Maris Teder-Laving, Caroline Hayward, Till Roenneberg, James F. Wilson, Fabiola Del Greco, Andrew A. Hicks, Chol Shin, Chang-Ho Yun, Seung Ku Lee, Andres Metspalu, Enda M. Byrne, Philip R. Gehrman, Henning Tiemeier, Karla V. Allebrandt, Rachel M. Freathy, Anna Murray, David A. Hinds, Timothy M. Frayling, Michael N. Weedon

**Affiliations:** 1 Genetics of Complex Traits, University of Exeter Medical School, Exeter, United Kingdom; 2 23andMe Inc., Mountain View, California, United States of America; 3 A9.com Inc, Palo Alto, California, United States of America; 4 Estonian Genome Center and Institute of Molecular and Cell Biology of University of Tartu, Estonian Biocentre, Tartu, Estonia; 5 Medical Research Council Human Genetics Unit, Institute of Genetics and Molecular Medicine, Edinburgh, Scotland; 6 Institute of Medical Psychology, Ludwig-Maximilians-University, Munich, Germany; 7 Usher Institute for Population Health Sciences and Informatics, University of Edinburgh, Edinburgh, Scotland; 8 Center for Biomedicine, European Academy of Bolzano, Bozen, Italy–affiliated Institute of the University of Lübeck, Lübeck, Germany; 9 Division of Pulmonary, Sleep and Critical Care Medicine, Department of Internal Medicine, Korea University Ansan Hospital, Ansan, Republic of Korea; 10 Institute of Human Genomic Study, College of Medicine, Korea University Ansan Hospital, Ansan, Republic of Korea; 11 Department of Neurology, Bundang Clinical Neuroscience Institute, Seoul National University Bundang Hospital, Seongnam, Republic of Korea; 12 The University of Queensland, Queensland Brain Institute, Brisbane, Australia; 13 Perelman School of Medicine of the University of Pennsylvania, Philadelphia, Pennsylvania, United States of America; 14 Department of Epidemiology, Erasmus Medical Center, Rotterdam, Netherlands; 15 Department of Psychiatry, Erasmus Medical Center, Rotterdam, Netherlands; National Cancer Institute, UNITED STATES

## Abstract

Disrupted circadian rhythms and reduced sleep duration are associated with several human diseases, particularly obesity and type 2 diabetes, but until recently, little was known about the genetic factors influencing these heritable traits. We performed genome-wide association studies of self-reported chronotype (morning/evening person) and self-reported sleep duration in 128,266 white British individuals from the UK Biobank study. Sixteen variants were associated with chronotype (*P*<5x10^-8^), including variants near the known circadian rhythm genes *RGS16* (1.21 odds of morningness, 95% CI [1.15, 1.27], *P* = 3x10^-12^) and *PER2* (1.09 odds of morningness, 95% CI [1.06, 1.12], *P* = 4x10^-10^). The *PER2* signal has previously been associated with iris function. We sought replication using self-reported data from 89,283 23andMe participants; thirteen of the chronotype signals remained associated at *P*<5x10^-8^ on meta-analysis and eleven of these reached *P*<0.05 in the same direction in the 23andMe study. We also replicated 9 additional variants identified when the 23andMe study was used as a discovery GWAS of chronotype (all *P*<0.05 and meta-analysis *P*<5x10^-8^). For sleep duration, we replicated one known signal in *PAX8* (2.6 minutes per allele, 95% CI [1.9, 3.2], *P* = 5.7x10^-16^) and identified and replicated two novel associations at *VRK2* (2.0 minutes per allele, 95% CI [1.3, 2.7], *P* = 1.2x10^-9^; and 1.6 minutes per allele, 95% CI [1.1, 2.2], *P* = 7.6x10^-9^). Although we found genetic correlation between chronotype and BMI (rG = 0.056, *P* = 0.05); undersleeping and BMI (rG = 0.147, *P* = 1x10^-5^) and oversleeping and BMI (rG = 0.097, *P* = 0.04), Mendelian Randomisation analyses, with limited power, provided no consistent evidence of causal associations between BMI or type 2 diabetes and chronotype or sleep duration. Our study brings the total number of loci associated with chronotype to 22 and with sleep duration to three, and provides new insights into the biology of sleep and circadian rhythms in humans.

## Author Summary

Numerous studies have identified links between too little or too much sleep and circadian misalignment with metabolic disorders such as obesity and type 2 diabetes. However, cause-and-effect is not easily determined, because of multiple confounding factors affecting both sleep patterns and disease risk. Using the first release of the UK Biobank study, which combines detailed measurements and questionnaire data with genetic data, we investigate the genetics of two self-report sleep measures, chronotype and average sleep duration, in 128,266 white British individuals. We replicate previous genetic associations and identify seven and two novel genetic variants influencing chronotype and sleep duration, respectively. Associated variants are located near genes implicated in circadian rhythm regulation (*RGS16*, *PER2*), near a serotonin receptor gene (*HTR6*) and another gene (*INADL*) encoding a protein thought to be important in photosensitive retinal cells, cells known to communicate with the brain’s primary circadian pacemaker. Using the genetic risk factors, we estimate the unconfounded causal associations of BMI and type 2 diabetes on sleep patterns (and vice versa) through Mendelian Randomisation. However, we find no evidence for causal associations in either direction. The full UK Biobank release of 500,000 individuals will boost our power to detect causal associations.

## Introduction

There are strong epidemiological associations between disrupted circadian rhythms, sleep duration and disease. A circadian rhythm refers to an underlying 24-hour physiological cycle that occurs in most living organisms. In humans, there are clear daily cyclical patterns in core body temperature, hormonal and most other biological systems [1]. These cycles are important for many molecular and behavioural processes. In particular, circadian rhythms are important in regulating sleeping patterns. While each individual has an endogenous circadian rhythm, the timing of these rhythms varies across individuals. Those with later circadian rhythms tend to sleep best with a late bedtime and late rising time and are often referred to as an “owl” or as an “evening” person. Those with earlier rhythms tend to feel sleepy earlier in the night and wake up early in the morning and are referred to as a “lark” or “morning” person. The remainder of the population falls in between these extremes. This dimension of circadian timing, or chronotype, is one behavioural consequence of these underlying cycles. Chronotype can be simply assessed by questionnaire and is considered a useful tool for studying circadian rhythms [2,3].

There is substantial evidence for a relationship between short sleep duration, poor quality sleep and obesity and type 2 diabetes [4,5]. Eveningness has been associated with poor glycaemic control in patients with type 2 diabetes independently of sleep disturbance [6] and with metabolic disorders and body composition in middle-aged adults [7]. There is evidence from animal models that disruption to circadian rhythms and sleep patterns can cause various metabolic disorders [8–10]. For example, mice homozygous for dominant negative mutations in the essential circadian gene, *Clock*, develop obesity and hyperglycaemia [10] and conditional ablation of the *Bmal1* and *Clock* genes in pancreatic islets causes diabetes mellitus due to defective β-cell function [9]. Despite this evidence, in humans the causal nature of the epidemiological associations between sleep patterns, circadian rhythms and obesity and type 2 diabetes is unknown. Identifying genetic variants associated with sleep duration and chronotype will provide instruments to help test the causality of epidemiological associations [11].

A previous genome-wide association study (GWAS) in 4,251 individuals identified a single genetic variant in *ABCC9* associated with sleep duration [12]. A subsequent GWAS meta-analysis including 47,180 individuals identified a single locus for sleep duration near *PAX8* [13]. Fifteen loci associated with chronotype were recently discovered by 23andMe [14] with 7 of these found to be in close proximity to known circadian rhythm regulation genes. The UK Biobank is a study of 500,000 individuals from the UK aged between 37 and 73 years with genome-wide SNP analysis and detailed phenotypic information, including chronotype and sleep duration (http://www.ukbiobank.ac.uk/). The UK Biobank study provides an excellent opportunity to identify novel genetic variants influencing chronotype and sleep duration which will provide insights into the biology of circadian rhythms and sleep and help test causal relationships between circadian rhythm and metabolic traits including obesity.

## Results

### Sixteen loci associated with chronotype in UK Biobank

**Fig 1:**
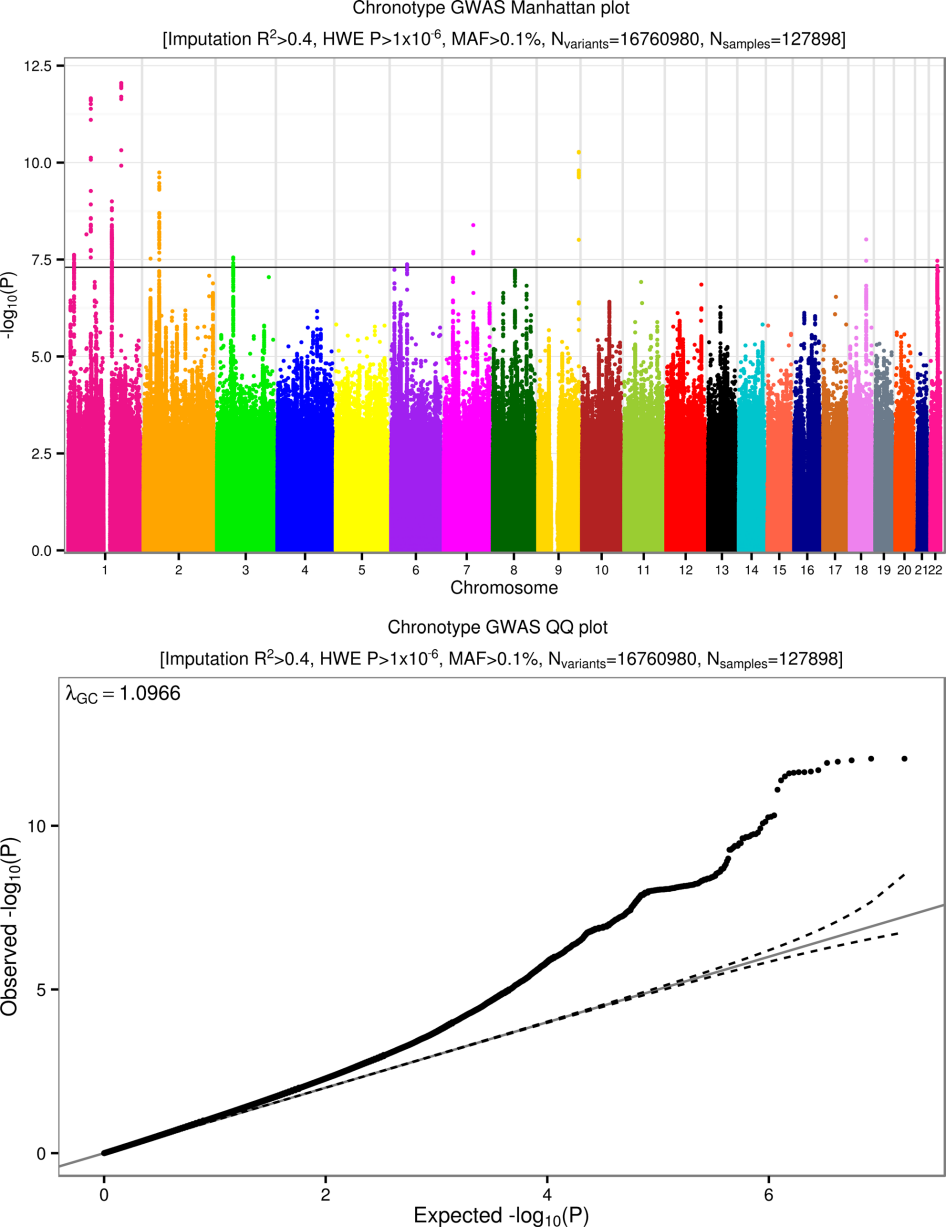
Manhattan and quantile-quantile (QQ) plots for chronotype. Summary information plots for inverse-normalised (self-report) chronotype score vs. ~16.8 million imputed genetic variants in 127,898 White British individuals in the UK Biobank study. The Manhattan plot (top) shows association test (-log_10_ *P*-value on the y-axis against physical autosomal location on the x-axis. The standard genome-wide significance cutoff of *P* = 5x10^-8^ is shown by the horizontal black line. Variants tested had imputation R^2^>0.4, a Hardy-Weinberg Equilibrium (HWE) *P*-value > 1x10^-6^ and minor allele frequency (MAF) > 0.1%. The QQ (quantile-quantile) plot (bottom) identifies some inflation (λ_GC_ = 1.097) but this is consistent with expected inflation from a highly polygenic trait in such a large sample size [15].

Using self-reported “morningness”, we generated a binary and a continuous chronotype score. We performed genome-wide association studies on 16,760,980 imputed autosomal variants. **Fig 1** presents the overall results for these GWAS. **Table 1** presents details of all 16 loci associated at *P*<5x10^-8^.

**Table 1:** Genetic variants associated with chronotype (as either a continuous or binary trait) at *P*<5x10^-8^ in the UK Biobank study. Variants highlighted in bold were not identified by the 23andMe study, those in italic did not reach genome-wide significance on meta-analysis and those not highlighted replicate previously reported loci from 23andMe. Genes listed are candidate or nearest genes within 250Kb of the lead SNP. Odds ratios correspond to risk of morningness over eveningness. Beta, OR and frequency refers to A1. Replication data is based on continuous data and as the replication beta is in different units to the discovery GWAS beta, a P-value meta-analysis was performed.

Variant	Chr:Pos (b37)	A1/A2	A1 Freq	GWAS Continuous Beta (SE)	GWAS Continuous P	GWAS Binary OR (SE)	GWAS Binary P	Replication Beta (SE)	Replication P	Combined P	Genes
rs516134	1:182,553,693	C/T	0.03	0.081 (0.011)	9.00E-13	1.21 (0.032)	3.00E-12	0.295 (0.035)	2.00E-17	7.00E-28	*RGS16*
rs11162296	1:77,700,196	G/C	0.84	-0.037 (0.005)	2.00E-12	0.93 (0.011)	1.00E-12	-0.097 (0.015)	2.00E-10	2.00E-21	*PIGK*, *AK5*
rs10157197	1:150,250,636	G/A	0.6	0.025 (0.004)	1.00E-09	1.05 (0.010)	5.00E-07	0.064 (0.011)	1.00E-08	6.00E-17	*PRPF3*, *TARS2*
rs372229746 *	7:102,158,815	G/A	0.55	0.028 (0.005)	4.00E-09	1.06 (0.012)	7.00E-07	0.068 (0.013)	4.00E-07	8.00E-15	*ORAI2*, *RASA4*
rs75804782	2:239,316,043	T/C	0.88	0.030 (0.006)	3.00E-07	1.09 (0.015)	4.00E-10	0.106 (0.018)	4.00E-09	1.00E-14	*PER2*
rs76899638	6:55,147,508	A/ATG	0.22	0.026 (0.005)	4.00E-08	1.05 (0.012)	2.00E-07	0.067 (0.014)	4.00E-06	8.00E-13	*HCRTR2 *
**rs77641763**	**9:140,265,782**	**C/T**	**0.88**	**0.039 (0.006)**	**5.00E-11**	**1.07 (0.015)**	**7.00E-09**	**0.065 (0.020)**	**2.00E-03**	**2.00E-12**	***EXD3***
**rs9961653**	**18:56,767,671**	**T/C**	**0.42**	**0.023 (0.004)**	**1.00E-08**	**1.04 (0.010)**	**1.00E-06**	**0.032 (0.012)**	**6.00E-03**	**7.00E-10**	***RAX*, *CPLX4*, *LMAN1***
**rs2050122**	**1:19,989,205**	**T/C**	**0.2**	**0.028 (0.005)**	**2.00E-08**	**1.06 (0.012)**	**3.00E-06**	**0.030 (0.014)**	**3.00E-02**	**1.00E-08**	***HTR6***
**rs70944707** *	**2:24,257,444**	**C/CT**	**0.23**	**0.030 (0.005)**	**3.00E-08**	**1.05 (0.013)**	**2.00E-05**	**0.035 (0.016)**	**3.00E-02**	**2.00E-08**	***FKBP1B***
**rs72720396**	**1:91,191,582**	**A/G**	**0.77**	**-0.025 (0.005)**	**1.00E-07**	**0.95 (0.010)**	**3.00E-08**	**-0.035 (0.014)**	**2.00E-02**	**2.00E-08**	***CALB1***
**rs12140153**	**1:62,579,891**	**G/T**	**0.9**	**0.039 (0.007)**	**7.00E-09**	**1.07 (0.017)**	**4.00E-06**	**0.043 (0.025)**	**8.00E-02**	**3.00E-08**	***INADL***
**rs1075265**	**2:54,354,927**	**C/G**	**0.48**	**-0.025 (0.004)**	**2.00E-10**	**0.95 (0.009)**	**4.00E-08**	**-0.010 (0.011)**	**4.00E-01**	**4.00E-08**	***PSME4*, *ACYP2***
*rs4821940*	*22*:*40*,*659*,*573*	*T/C*	*0*.*45*	*0*.*022 (0*.*004)*	*3*.*00E-08*	*1*.*05 (0*.*010)*	*4*.*00E-08*	*0*.*006 (0*.*011)*	*6*.*00E-01*	*5*.*00E-06*	*SGSM3*
*rs12635074*	*3*:*55*,*982*,*416*	*T/G*	*0*.*68*	*-0*.*023 (0*.*004)*	*3*.*00E-08*	*0*.*96 (0*.*009)*	*2*.*00E-06*	*-0*.*002 (0*.*012)*	*8*.*00E-01*	*1*.*00E-05*	*ERC2*
*rs192534763*	*8*:*36*,*202*,*946*	*T/C*	*0*.*99*	*0*.*100 (0*.*021)*	*3*.*00E-07*	*1*.*25 (0*.*057)*	*2*.*00E-08*	*-0*.*005 (0*.*055)*	*9*.*00E-01*	*1*.*00E-04*	*UNC5D*

* Proxies used for replication cohort: rs4729854 for rs372229746 (r^2^ = 0.33), and rs12621152 for rs70944707 (r^2^ = 0.33).

### Replication and validation of UK Biobank chronotype associations

Analysing UK Biobank data with that from 23andMe provides evidence that at least 13 of the 16 are associated with chronotype. Thirteen of the chronotype signals remained at *P*<5x10^-8^ in a meta-analysis including UK Biobank and 89,283 individuals from 23andMe [14], of which 11 reached *P*<0.05 in the same direction in 23andMe alone, and 15 of the 16 UK Biobank signals were in the same direction (binomial *P* = 0.0002) (**Table 1**). We also attempted to validate the associations in 6,191 European-Ancestry from the Chronogen consortium and 2,532 Korean Ancestry individuals from the Insomnia, Chronotype and sleep EEG (ICE) consortium that used “Gold standard” chronotype questionnaire (Munich Chronotype Questionnaire–MCTQ and Morningness-Eveningness Questionnaire—MEQ). Given the sample size of 5% of the discovery UK Biobank study we assessed directional consistency rather than testing for replication P-values <0.05 or 0.05/16. In the European-Ancestry individuals 11 of the 16 signals were represented. Nine of these 11 variants had the same direction of effect as the discovery UK Biobank cohort (binomial test *P* = 0.03) and one replicated at Bonferroni significance (rs12140153, *P* = 0.003). In the Korean study, 9 signals were represented, four of which had the same direction of effect as the discovery UK Biobank cohort (binomial test *P* = 1.00). The level of directional consistency in these two smaller studies is consistent with what would be expected in cohorts <5% the size of our discovery cohort.

### Replication of previously reported chronotype associations

A 23andMe study recently identified 15 loci associated with chronotype [14]. All of the 15 signals were replicated in our study with *P*<0.05 in the same direction and had meta-analysis *P*<5x10^-8^ (**S1 Table**). We performed a conditional analysis of our lead chronotype variants by adjusting for the 15 known signals (**S2 Table**), in order to identify which of our loci coincided with those of Hu *et al*. [14]. Seven of our 13 replicated signals remained associated at *P*<5x10^-8^ (see **Table 1**). The addition of these 7 loci brings the number associated with chronotype to 22 (full list in **S3 Table**).

### The chronotype-associated variants occur near genes known to be important in photoreception and circadian rhythms

**Fig 2:**
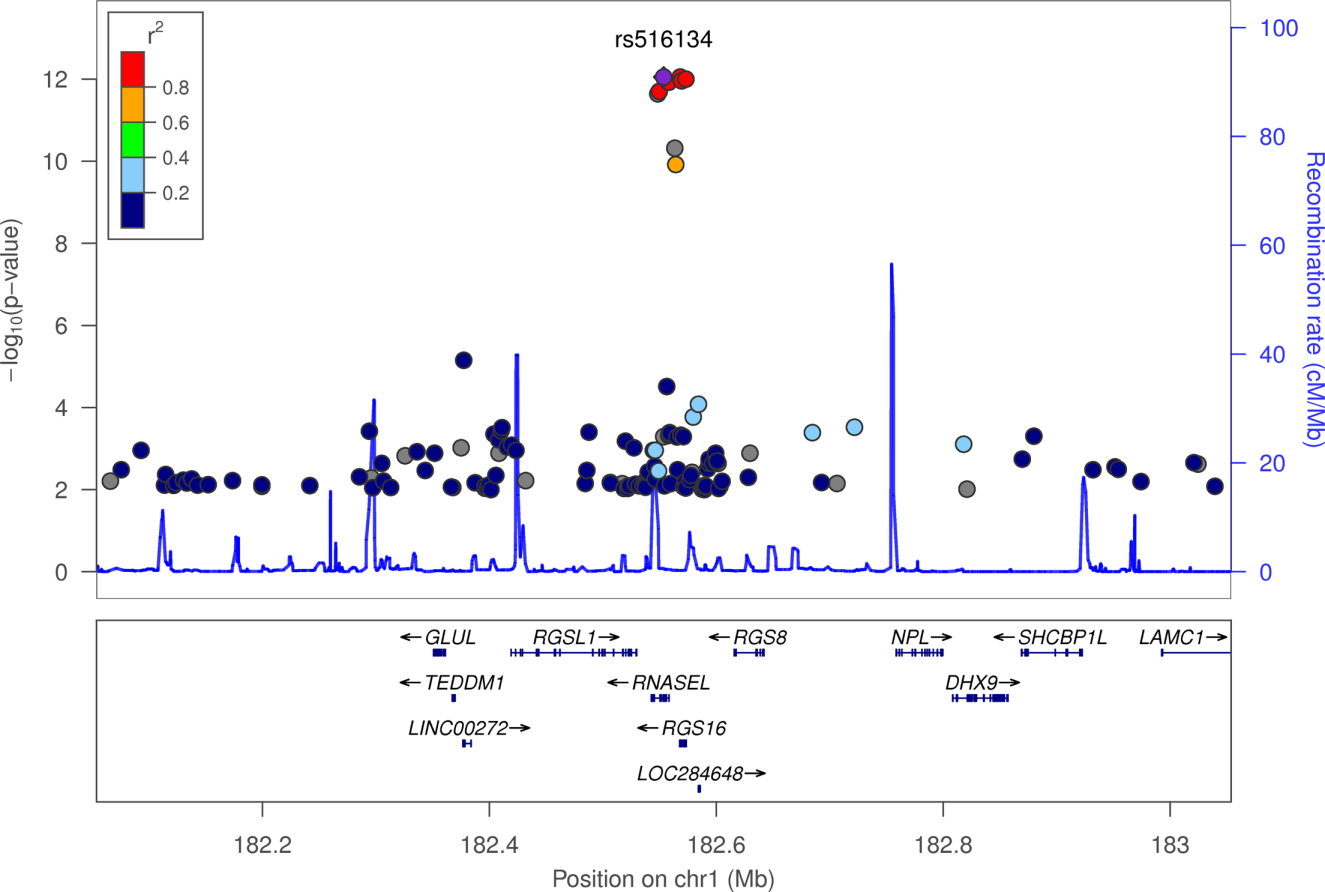
LocusZoom plot of chronotype associations in the *RGS16* locus. The plot displays -log_10_ *P*-value on the y-axis and physical position on the x-axis. Points identify individual variants whose colour indicates their LD r^2^ with lead variant rs516134. The blue line indicates pre-calculated recombination rates (in cM/Mb) at each position. Variants with association *P*-values > 0.01 were omitted for clarity.

**Fig 3:**
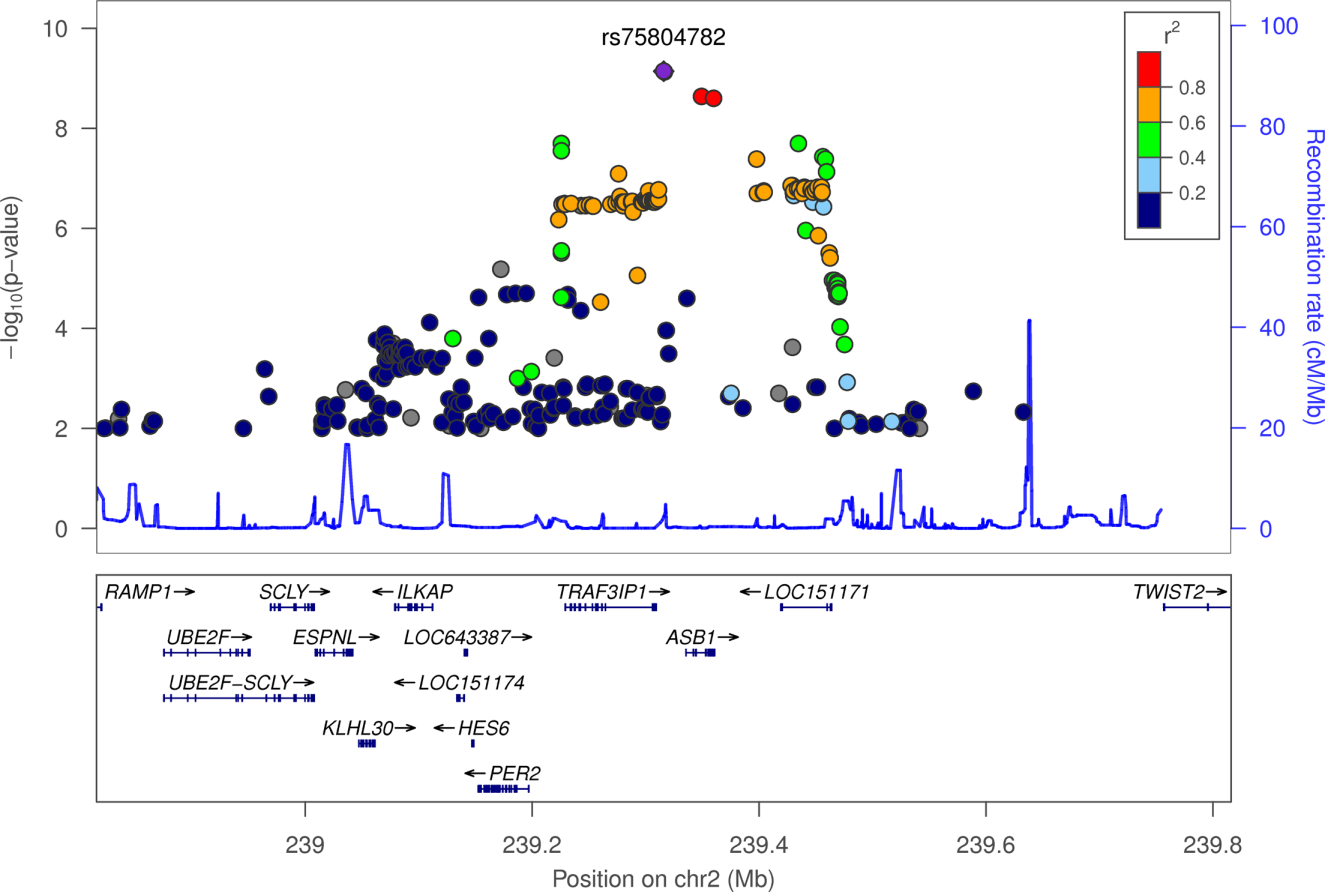
LocusZoom plot of chronotype associations in the *PER2* locus. Variants are coloured by their LD r^2^ with lead variant rs75804782 and those with association *P*-values > 0.01 were omitted for clarity.

The variant most strongly associated with chronotype, rs516134 (OR for morningness = 1.21, 95% CI [1.16, 1.26], binary *P* = 3.7x10^-12^, continuous *P* = 8.9x10^-13^) occurs near *RGS16*, which is a regulator of G-protein signalling and has a known role in circadian rhythms [16] (**Table 1 and Fig 2**). Another signal occurs near *PER2* (lead variant rs75804782, OR = 1.09, 95% CI [1.06, 1.12], binary *P* = 7.2x10^-10^, continuous *P* = 3.2x10^-7^; **Fig 3**). *PER2* is a well-known regulator of circadian rhythms [17–22] and contains a variant, rs75804782, recently shown to be associated with iris formation [23] that is in LD (r^2^ = 0.65, D’ = 0.97) with our reported lead SNP. As there is a reported link between season and reported chronotype [24], we carried out a sensitivity analysis in which we adjusted for month of attendance (to assessment centre); all associations remained genome-wide significant for the reported variants. We tested for enrichment of specific biological and molecular pathways using MAGENTA (Meta-Analysis Gene-set Enrichment of variaNT Associations) [25] but none had a clear link to circadian rhythms (**S4 Table**).

### Three loci associated with sleep duration

**Fig 4:**
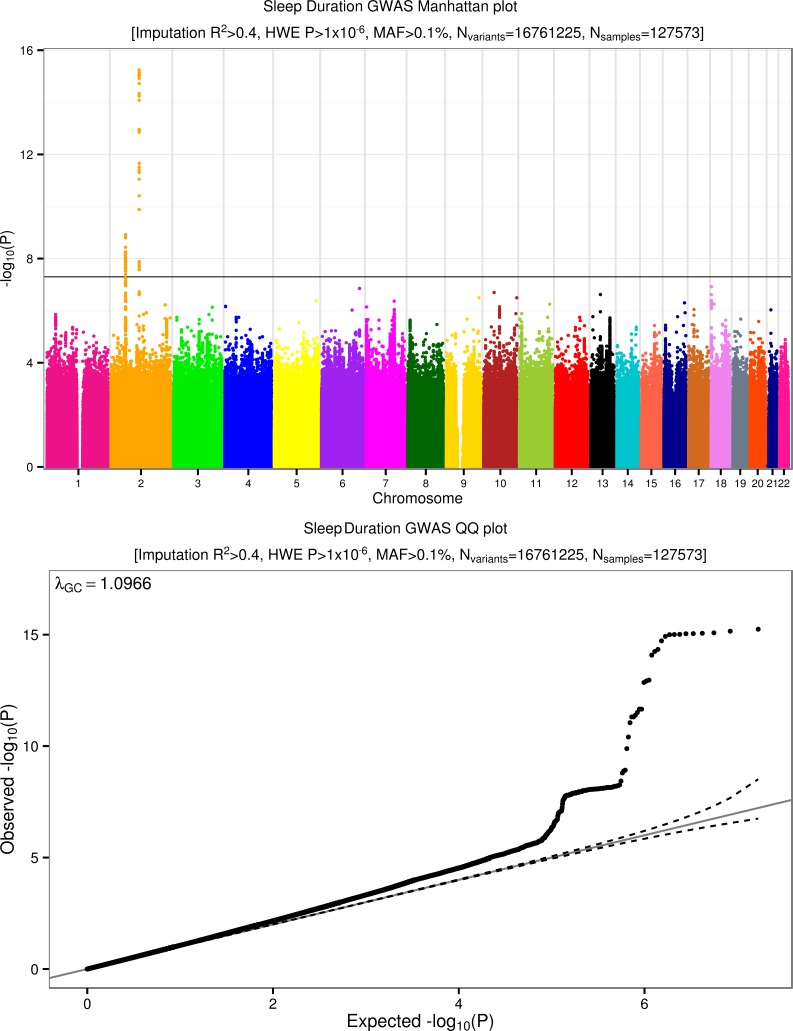
Manhattan and quantile-quantile (QQ) plots for chronotype. Summary information plots for inverse-normalised (self-report) Sleep Duration vs. ~16.8 million imputed genetic variants in 127,573 White British individuals in the UK Biobank study. The Manhattan plot (top) shows association test (-log_10_ *P*-value on the y-axis against physical autosomal location on the x-axis with the standard genome-wide significance cutoff of *P* = 5x10^-8^ shown by the horizontal black line. Variants tested had imputation R^2^>0.4, a Hardy-Weinberg Equilibrium (HWE) *P*-value > 1x10^-6^ and minor allele frequency (MAF) > 0.1%. The Sleep Duration QQ plot (bottom) identifies some inflation (λ_GC_ = 1.097) but, as with Chronotype, this is consistent with expected inflation from a highly polygenic trait in such a large sample size [15].

**Fig 5:**
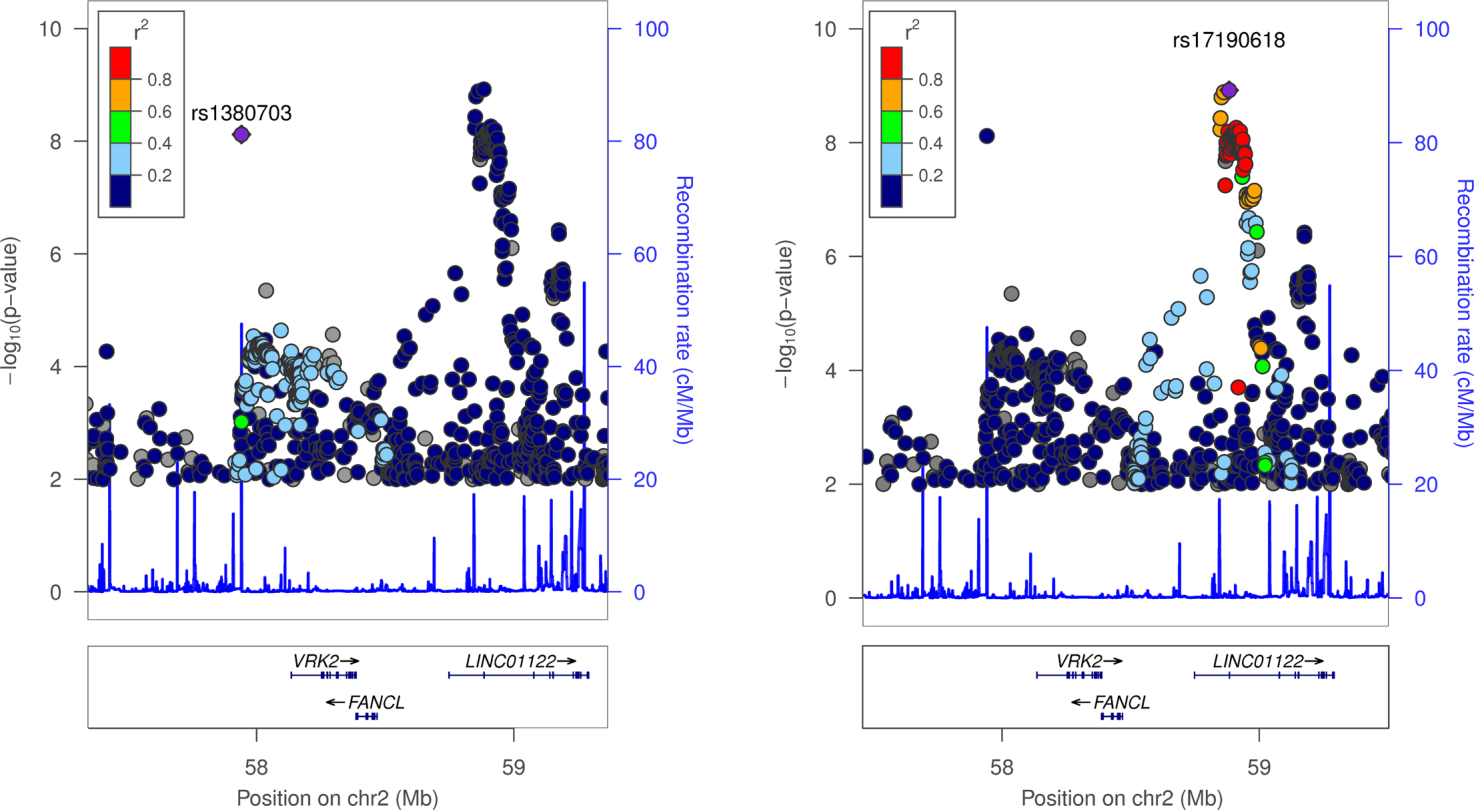
LocusZoom plots of sleep duration associations in the *VRK2* locus. Both plots show the same locus but each highlights a different lead variant: rs1380703 (left) and rs17190618 (right). Variants with association *P*-values > 0.01 were omitted for clarity. The two leads variants represent separate signals.

We performed genome-wide association studies on a binary sleep phenotype and a continuous sleep duration score for 16,761,225 imputed variants. **Fig 4** presents the overall results for these GWAS. Three loci reached genome-wide significance. The most strongly associated variant was rs62158211 with an average 2.6 minute (95% CI [1.9, 3.2], *P* = 5.7x10^-16^) per-allele change in sleep duration and occurs at the previously reported association signal near *PAX8* [13]. We identified two, novel, conditionally independent, signals that were located ~900kb apart, one upstream and the other downstream of *VRK2*. The downstream variant, rs17190618, has an average per allele effect of 2.0 minutes (95% CI [1.3, 2.7], *P* = 1.2x10^-9^) on sleep duration. The upstream variant, rs1380703 (which is not correlated with rs17190618, r^2^ = 0.002), has an average per allele effect of 1.6 minutes (95% CI [1.1, 2.2], *P* = 7.6x10^-9^) on sleep duration. On adjusting for month of assessment, we saw similar associations for both rs62158211 (*P* = 3x10^-16^) and rs1380703 (*P* = 6x10^-9^), with no change for rs17190618. **Table 2** shows the three sleep duration loci and their lead variants. **Fig 5** shows locus zoom plots of the *VRK2* association signals. We did not replicate the association of a previously reported variant in *ABCC9* [12] with sleep duration (rs11046205, 0.1mins, 95% CI [-0.6, 0.7], *P* = 0.83).

**Table 2:** Three loci associated with sleep duration and their lead variants. Genes listed are candidate genes at each locus. Beta, OR and frequency refers to A1. Because the replication beta is in different units to the discovery GWAS beta, a P-value meta-analysis was performed. Beta units are in hours.

Variant	Chr:Pos (b37)	A1/A2	A1 Freq	GWAS Continuous Beta (SE)	GWAS Continuous P	GWAS Binary OR (SE)	GWAS Binary P	Replication Beta (SE)	Replication P	Combined P	Gene
rs62158211	2:114,106,139	G/T	0.79	-0.039 (0.005)	6E-16	0.94 (0.011)	1E-07	-0.053 (0.009)	4E-9	2E-23	PAX8
rs17190618	2:58,882,765	A/T	0.84	-0.033 (0.005)	1E-09	0.96 (0.013)	3E-04	-0.035 (0.011)	1E-3	5E-12	VRK2
rs1380703	2:57,941,287	A/G	0.62	0.025 (0.004)	8E-09	1.06 (0.011)	8E-08	0.021 (0.008)	1E-2	3E-10	VRK2

### Replication of novel sleep duration hits

To replicate the two novel sleep duration hits we used data from 47,180 individuals from a published study [13]. The variant rs17190618 replicated with effect size = 2.1 minutes (95% CI [0.8, 3.3], *P* = 0.001, meta-analysis *P* = 5x10^-12^). The variant rs1380703 replicated with effect size = 1.3 minutes (95% CI [0.3, 2.2], *P* = 0.01, meta-analysis *P* = 3x10^-10^).

### Sleep duration and chronotype are heritable and genetically correlated with BMI and psychiatric disease

Using LD-score regression we estimated the heritability of chronotype and sleep duration within UK Biobank to be 0.12 (0.007), and 0.07 (0.007), respectively. There was no evidence of a genetic correlation between sleep duration and chronotype (rG = 0.0177, *P* = 0.70). Chronotype was nominally genetically correlated with BMI (rG = 0.056, *P* = 0.048), but not Type 2 diabetes (rG = 0.004, *P* = 0.99). As the relationship between sleep duration with BMI and risk of T2D is U-shaped (see **S1 Fig**), we defined two further binary phenotypes; undersleepers (<7 vs. 7–8 hours) and oversleepers (>8 vs. 7–8 hours). There was a strong genetic correlation between undersleeping and BMI (rG = 0.147, *P* = 1x10^-5^), but not T2D (rG = 0.022,*P* = 0.79). There was also a genetic correlation between oversleeping and both BMI (rG = 0.097, *P* = 0.039) and T2D (rG = 0.336, *P* = 0.001). We also performed LD-score regression analyses against a range of other diseases and traits where GWAS summary statistics are publicly available (**S5 Table**). Schizophrenia was genetically correlated (after adjusting for the number of tests) with hours slept (rG = 0.26, *P* = 5x10^-10^), oversleeping (rG = 0.35, *P* = 6x10^-8^), undersleeping (rG = -0.14, *P* = 2x10^-3^) and chronotype (rG = -0.12, *P* = 2x10^-4^).

### Mendelian randomisation analyses provide no consistent evidence that higher BMI affects self-reported morningness or vice-versa

The genetic correlations we observed provide general estimates that capture pleiotropic variants (those that affect both traits through different pathways) and associations that are secondary to a variant affecting a trait that causally influences the second trait. Using a genetic risk score of 69 known BMI variants [26] (listed in **S6 Table**) as an instrumental variable, we next performed Mendelian randomisation analyses in the UK Biobank study to test the potential causal role of BMI in chronotype and sleep. Instrumental variables analyses using variants and their effect sizes identified by previous studies [26] provided no consistent evidence that BMI causally affects self-reported “morningness” (**S7 Table**). Association statistics of the BMI variants with chronotype are given in **S6 Table**. We repeated these analyses using a genetic risk score consisting of 55 type 2 diabetes SNPs [27] and did not find any evidence of causality. Performing the reciprocal Mendelian randomization analysis using a genetic risk score of the 13 replicated chronotype variants, with effect sizes obtained from 23andMe, we found no consistent evidence in the UK Biobank data that morningness or eveningness leads to higher BMI (**S7 Table**). Association of the chronotype-associated variants with BMI are given in **S8 Table**.

### No evidence that BMI and type 2 diabetes are causally associated with sleep duration

Using the same genetic risk score of 69 known BMI variants as an instrument, we saw no consistent evidence that higher BMI increased an individual’s likelihood of being an undersleeper (IVreg2 *P* = 0.95, IVW *P* = 0.04) or an oversleeper (IVreg2 *P* = 0.29, IVW *P* = 0.62) in the UK Biobank data (**S7 Table**). Because there were only three genetic variants of small effect associated with sleep duration, we did not perform any Mendelian Randomisation analyses of sleep on BMI or type 2 diabetes risk.

## Discussion

We performed a genome-wide association study of sleep duration and morningness in 128,266 individuals from the UK Biobank study. We discovered and replicated two novel loci associated with sleep duration. Through replication in a study of 89,823 individuals from 23andMe we found 13 loci associated with chronotype at *P*<5x10^-8^. Together with a recent study from 23andMe [14] this takes the number of replicated loci for being a morning person to 22 (7 not reported in the 23andMe study). These loci occur in or near circadian rhythm and photoreception genes and provide new insights into circadian rhythm and sleep biology and their links to disease.

The two novel sleep duration association signals occur upstream and downstream of *VRK2* (*vaccinia related kinase 2*). VRK2 is a serine/threonine kinase important in several signal transduction cascades, and variants near *VRK2* are associated with schizophrenia [28] and epilepsy [29]. The two sleep duration variants we identified do not represent the same signals as those associated with schizophrenia at genome wide significance but one is associated with schizophrenia (based on publically available data from the schizophrenia genetics consortium (rs1380703) at *P* = 2x10^-5^, with the allele associated with more sleep being associated with higher risk of schizophrenia). Furthermore, the variants associated with epilepsy and schizophrenia at genome wide significance in previous studies are associated with sleep duration in UK Biobank (epilepsy lead variant rs2947349 [29], *P* = 2x10^-5^ and schizophrenia lead variant [28] rs11682175 *P* = 3x10^-5^) but did not reach genome wide significance. We also observed genetic correlation between sleep duration and schizophrenia using LD-score regression (rG = 0.26, *P* = 5x10^-10^). Further work is required to determine whether variation in *VRK2* either has independent associations with both sleep and schizophrenia or whether there is some causal link between sleep duration and pattern and schizophrenia and epilepsy.

Several of the loci that we identified as associated with chronotype contain genes that have a known role in circadian rhythms. The most strongly associated variant, rs516134, occurs 20kb downstream of *RGS16* (regulator of G protein signalling 16). *RGS16* has recently been shown to have a key role in defining 24 hour rhythms in behaviour [16]. In mice, gene ablation of *Rgs16* lengthens the circadian period of behavioural rhythm [16]. By temporally regulating cAMP signalling, Rgs16 has been shown to be a key factor in synchronising intercellular communication between pacemaker neurons in the suprachiasmatic nucleus (SCN), the centre for circadian rhythm control in humans.

The association signal with lead SNP rs75804782 occurs ~100kb upstream of *PER2* (*Period 2*). Per2 is a key regulator of circadian rhythms and is considered one of the most important clock genes, and, under constant darkness, *Per2* knockout mice show arrhythmic locomotor activity [17–22]. This locus also contains a variant that has recently been shown to be associated with iris furrow contractions [23]. Our signal is very likely to represent the same association and suggests a link between iris function and chronotype (rs75804782 has an LD r^2^ = 0.65 and D’ = 0.97 with the reported lead SNP, rs3739070). Larsson *et al*. [23] suggest *TRAF3IP1* as the most likely candidate gene at the locus because of its critical role in the cytoskeleton and neurogenesis. Further work is needed to elucidate whether the chronotype association at this locus acts through *PER2* or *TRAF3IP1*.

Four of the 22 chronotype loci had missense variants in LD (r^2^>0.8) with the lead variant (*RGS16*, *EXD3*, *INADL* and *HCRTR2*; see **S9 Table**). The *INADL* variant association is particularly interesting as INADL (InaD-like) encodes a protein that has been thought to be important in organising and maintaining the “intrinsically photosensitive retinal ganglion cells”, cells that are known to communicate directly with the suprachiasmatic nucleus, the primary circadian pacemaker in mammals [30]. This is compelling evidence that *INADL* is involved in the human circadian rhythm pathway.

Several of the variants associated with chronotype are also associated with BMI and we found genetic correlation between chronotype and sleep duration and BMI. There is substantial evidence for a role of sleep disruption and circadian rhythms in metabolic disease [1]. Data from animal models and epidemiology provide strong evidence that sleep quality or disrupted circadian rhythms can cause metabolic diseases including obesity and type 2 diabetes [4–6,8–10]. Our Mendelian Randomisation analyses provided no consistent evidence for a role of higher BMI leading to increased self-reported morningness. These Mendelian Randomisation results are consistent with those from the recent study from 23andMe [14].

There are some important limitations to our study. First, chronotype and sleep duration were self-reported and are subject to reporting bias (e.g. obese individuals may be more likely to falsely claim to be morning people). Second, whilst we did not find any evidence that overall chronotype or sleep duration causally lead to obesity or type 2 diabetes, it is possible that sub-pathways of genes involved in, for example, feeding behaviour may be important in both obesity and chronotype regulation. Third, the identified variants only account for a small amount of the variation in chronotype and sleep duration and we therefore had limited power to detect an effect of these variants on BMI or type 2 diabetes risk. The availability of the full UK Biobank study of 500,000 will provide further insight into this relationship.

In conclusion, we have identified novel genetic associations for chronotype and sleep duration. The chronotype loci cluster near genes known to be important in determining circadian rhythms and will provide new insights into circadian regulation. Our results provide new insights into circadian rhythm and sleep biology and their links to disease.

## Materials and Methods

### Ethics statement

This study was conducted using the UK Biobank resource. Details of patient and public involvement in the UK Biobank are available online (www.ukbiobank.ac.uk/about-biobank-uk/ and https://www.ukbiobank.ac.uk/wp-content/uploads/2011/07/Summary-EGF-consultation.pdf). No patients were specifically involved in setting the research question or the outcome measures, nor were they involved in developing plans for recruitment, design, or implementation of this study. No patients were asked to advise on interpretation or writing up of results. There are no specific plans to disseminate the results of the research to study participants, but the UK Biobank disseminates key findings from projects on its website.

### Discovery samples

We used 128,266 individuals of British descent from the first UK Biobank genetic data release (see http://biobank.ctsu.ox.ac.uk). British-descent was defined as individuals who both self-identified as white British and were confirmed as ancestrally Caucasian using principal components analyses (http://biobank.ctsu.ox.ac.uk). Of these individuals, 120,286 were classified as unrelated, with a further 7,980 first- to third-degree relatives of these. As the association tests were carried out in BOLT-LMM [31], which adjusts for relationships between individuals and corrects for population structure, we included all 128,266 related white British individuals in the association analyses.

### Genotyping and quality control

We used imputed variants provided by the UK Biobank. Details of the imputation process are provided at the UK Biobank website (see http://biobank.ctsu.ox.ac.uk). For this study we only included the ~16.8M imputed variants with an imputation R^2^ ≥ 0.4, MAF ≥ 0.001 and with a Hardy–Weinberg equilibrium *P*>1x10^-5^.

### Phenotypes

#### Chronotype

UK Biobank provides a single measure of Chronotype, from which we produced a continuous and a dichotomous phenotype. Chronotype (or morningness) is a self-reported measure and asks individuals to categorise themselves as “Definitely a ‘morning’ person”, “More a ‘morning’ than ‘evening’ person”, “More an ‘evening’ than a ‘morning’ person”, “Definitely an ‘evening’ person” or “Do not know”, which we coded as 2, 1, -1, -2 and 0 respectively, in our raw continuous “score”. Individuals had the option not to answer; these individuals were set to missing. We then produced a normally distributed phenotype by adjusting the raw phenotype for age, gender and study centre (categorical) and inverse normalising the resulting residuals. The dichotomous chronotype trait defines morning people (“Definitely a ‘morning’ person” and “More a ‘morning’ than ‘evening’ person”) as cases and evening people (“Definitely an ‘evening’ person” and “More an ‘evening’ than a ‘morning’ person”) as controls. All other individuals are coded as missing. All results reported for continuous chronotype refer to the inverse-normalised residualised chronotype “score”. For interpretable results, however, we report effect sizes using the odds ratios of the dichotomous chronotype phenotype. A total number of 127,898 and 114,765 individuals were available with non-missing continuous and binary chronotype phenotypes, respectively, for the association tests; for the Mendelian Randomisation this became 119,935 and 107,634 respectively.

#### Sleep duration

The UK Biobank also provides self-reported “sleep duration”, in which individuals were asked to provide the average number of hours slept in a 24-hour period. The phenotype was derived by first excluding individuals reporting greater than 18 hours sleep, then adjusting for age, gender and study centre (categorical) and obtaining the model residuals and finally inverse-normalising to assure a normally distributed phenotype. When reporting results for the continuous sleep duration phenotype, we are referring to the inverse-normalised phenotype, though we report effect sizes of the residualised phenotype to allow easier interpretation of results. There were 127,573 individuals with reported sleep duration available for the association tests, with 119,647 available for the MR analyses.

#### “Oversleepers” and “undersleepers”

These two dichotomous phenotypes share the same set of controls; those individuals that reported sleeping either 7 or 8 hours (81,204 individuals). In oversleepers, cases (10,102 individuals) are those reporting 9 or more hours sleep on average, whereas undersleeper cases (28,980 individuals) are those reporting 6 or fewer hours.

#### BMI

The UK Biobank provided a BMI (weight (kg)/height^2^) measurement and an estimate based on electrical impedance analyses. To help avoid reporting error we excluded individuals with significant differences (>4.56 SDs) between these two variables where both were available. If only one of these measurements was available this was used. We corrected BMI by regressing age, sex, study centre, and the first 5 within-British principal components and taking residual values. We then inverse normalised the residuals. A total of 119,684 white-British individuals with BMI and genetic data were available for the Mendelian Randomisation analyses.

#### Type 2 diabetes

Individuals were defined as having T2D if they reported either T2D or generic diabetes at the interview stage of the UK Biobank study. Individuals were excluded if they reported insulin use within the first year of diagnosis. Individuals reportedly diagnosed under the age of 35 years or with no known age of diagnosis were excluded, to limit the numbers of individuals with slow-progressing autoimmune diabetes or monogenic forms. Individuals diagnosed with diabetes within the last year of this study were also excluded as we were unable to determine whether they were using insulin within this time frame. A total of 4,040 cases and 113,735 controls within the white British subset of UK Biobank were identified with genetic data available.

### Genome-wide association analysis

To perform the association tests, we used BOLT-LMM [31] to perform linear mixed models (LMMs) in the 128,266 individuals. We used BOLT-LMM as it adjusts for population structure and relatedness between individuals whilst performing the association tests with feasible computing resources. As it adjusts for population structure and relatedness, it allowed us to include the additional 7,980 related individuals and therefore improved our power to detect associations. To calculate the relationships between individuals, we provided BOLT-LMM a list of 328,928 genotyped SNPs (MAF>5%; HWE *P*>1x10^-6^; missingness<0.015) for the individuals included in the association analysis and used the 1000 Genomes LD-Score table provided with the software.

As the continuous phenotypes were derived by adjusting for age, gender and study centre, the LMM only included chip (BiLEVE vs. UKBiobank arrays) as a covariate at run-time (see http://www.ukbiobank.ac.uk/wp-content/uploads/2014/04/UKBiobank_genotyping_QC_documentation-web.pdf). The binary phenotypes were unadjusted and so included age, gender and chip at run-time. BOLT-LMM reported no improvement of the non-infinitesimal mixed model test over the standard infinitesimal test and so all association results reported in this paper are for the infinitesimal model [31].

### Chronotype replication analyses

Participants (N = 89,283) were from the customer base of 23andMe, Inc. The descriptions of the samples, genotyping and imputation are in [14]. Of the 16 chronotype-associated variants for which we attempted replication, 10 were available from imputation from the 1000 Genomes imputation panel phase 1 pilot. An additional 4 were imputed from the phase 1 version 3 1000 Genomes imputation panel. The final two could not be imputed. We used http://analysistools.nci.nih.gov/LDlink/ to find proxies—the best available were rs4729854 for rs372229746 (r^2^ = 0.33), and rs12621152 for rs70944707 (r^2^ = 0.33). We meta-analysed *P*-values from the discovery and replication samples using sample size weighting implemented in METAL [32].

### Chronotype validation analyses

Genotypes consisting of both directly typed and imputed SNPs were used for the individual GWAS [12]. To avoid over-inflation of test statistics due to population structure or relatedness, we applied genomic control for the independent studies and meta-analysis. Linear regression for associations with normalised chronotype was performed (see [12] for packages used) under an additive model, with SNP allele dosage as predictor and with age, age^2^, gender, normalised sleep duration, season of assessment (dichotomized based on time of the year, and day-light savings time–DST or standard zone time assessments) as covariates. A fixed-effects meta-analysis was conducted with GWAMA (Genome-Wide Association Meta-Analysis) [33] using the inverse-variance-weighted method and low imputation quality (Rsq/proper_info < 0.3) were dropped from the meta-analysis.

### Pathway and functional annotation analyses

Pathway analyses were carried out in MAGENTA[25] using all available libraries provided with the software. We included all imputed variants with association *P*<1x10^-5^ from the continuous chronotype trait. For the results presented in **S4 Table**, we used gene upstream and downstream limits of 250Kb, excluded the HLA region (default setting) and set the number of GSEA (Gene Set Enrichment Analysis) [34,35] permutations at 10,000 (default). We used HaploReg v4.1[36] to annotate any coding variants within LD r^2^ > 0.8 of the lead variant at each locus.

### LocusZoom plots

LocusZoom plots (**Figs 2**, **3** and **5**) were created using the LocusZoom tool [37] (found at http://locuszoom.sph.umich.edu/locuszoom/) by uploading summary statistics from the Chronotype and sleep duration GWAS. For the background LD structure, we selected the “1000 Genomes Nov 2014 EUR” panel.

### Genetic correlation analyses

Genetic correlations (see [38] for methodology) between traits were calculated using the LD Score Regression software LDSC (available at https://github.com/bulik/ldsc/) [39]. Summary statistics of our traits outputted by BOLT-LMM were first “munged”, a process that converts the summary statistics to a format that LDSC understands and aligns the alleles to the Hapmap 3 reference panel, removing structural variants and multi-allelic and strand-ambiguous SNPs. Genetic correlations were then calculated between our phenotypes and a set of 101 phenotypes for which summary statistics are publicly available (full list in **S5 Table**). We used precomputed LD structure data files specific to Europeans of HAPMAP 3 reference panel, obtained from (http://www.broadinstitute.org/~bulik/eur_ldscores/) as suggested on the LDSC software website.

### Mendelian Randomisation IV analysis

The 13 variants in **Table 1** which reached *P*<5x10^-8^ in combined analyses were used as chronotype instruments in the Mendelian Randomisation analyses. Where binary and continuous traits shared a locus, we selected the top variant of the continuous trait over that of the binary. For loci that reach GW-significance in the binary trait only, we selected the top variant but used the effect size from the continuous trait.

To test for a causal effect of BMI on chronotype and sleep-duration, we selected 69 of 76 common genetic variants that were associated with BMI at genome wide significance in the GIANT consortium in studies of up to 339,224 individuals (**S6 Table**) [26]. We limited the BMI SNPs to those that were associated with BMI in the analysis of all European ancestry individuals and did not include those that only reached genome-wide levels of statistical confidence in one sex or one stratum only. Variants were also excluded if known to be classified as a secondary signal within a locus. Three variants were excluded from the score due to potential pleiotropy (rs11030104 [*BDNF* reward phenotypes], rs13107325 [*SLC39A8* lipids, blood pressure], rs3888190 [*SH2B1* multiple traits]), three due to being out of HWE (rs17001654, rs2075650 and rs9925964) and the last variant due to not being present in the imputed data (rs2033529).

For testing reverse causality of type 2 diabetes on our sleep phenotypes, we used 55 of 65 common variants (listed in **S6 Table**) known to be associated with type 2 diabetes at genome wide significance in a meta-analysis of 34,840 cases and 114,981 [27], excluding those known or suspected to be pleiotropic.

We performed the Mendelian Randomisation analysis two ways; firstly using instrumental variables (IV) using STATA’s “IVreg2” function [40] and secondly through the inverse-variance weighted (IVW) and MR-Egger methods described in [41]. Analyses were performed in STATA 13.1 (StataCorp. 2013. Stata Statistical Software: Release 13. College Station, TX: StataCorp LP.).

In the instrumental variables method, we generated genetic risk scores (GRS) for BMI and type 2 diabetes using the published list of associated variants and their respective betas. For Chronotype, we generated a GRS using the thirteen replicated variants and their respective betas from 23andMe summary statistics. Using the IVreg2 command, we performed two-stage least squares estimation to calculate the effect of predicted exposure (through the GRS) on the continuous outcome traits. For binary outcomes (type 2 diabetes, undersleeper and oversleeper), we manually carried out the two-stage process by regressing the exposure trait on its GRS and storing both predicted values and residuals. We then used these predicted values and residuals as independent variables in a logistic regression where the dependent variable was the binary outcome.

The inverse-variance weighted (IVW) method is equivalent to a meta-analysis of the associations of the individual instruments and uses associations between the instruments and both the exposure and the outcome to estimate the additive effect of the instruments combined [41]. The MR-Egger method is a modification to the IVW method that allows the inclusion of “invalid” instruments (i.e. those that don't satisfy all three conditions), by performing Egger regression using the summary data of the variants. The IVW and Egger methods operate under the assumption that all instruments are valid, in that they satisfy the three IV conditions: the genetic variants are 1) independent of confounders, 2) associated with the exposure and 3) independent of the outcome. The MR-Egger method, however, accounts for the fact that genetic variants could be pleiotropic and may influence the outcome via pathways other than through the exposure and therefore the resulting association between genetic instruments and the outcome should not be biased by invalid instruments and pleiotropy. The MR-Egger method was used purely as a sensitivity test for the IVW method and so MR-Egger results were not considered if the IVW result did not reach nominal significance.

For the IVW and MR-Egger methods, associations of genetic instruments (variants) with both exposure and outcome phenotypes were generated in STATA by regressing the phenotype against the instrument while adjusting for covariates. As a further sensitivity test, we also repeated these analyses by replacing exposure phenotype-variant associations with their respective published betas and found only slight differences in betas and *P*-values, though all exposure-outcome associations remained non-significant.

## Supporting Information

S1 Table Replication of 23andMe lead GW-significant variants (see Hu *et al*., 2016) [14]. The combined *P*-value was generated by meta-analysing the 23andMe and UKB continuous chronotype *P*-values in the meta-analysis software METAL.
(XLSX)

S2 Table Conditional analysis to identify loci independent to those reported in Hu et al., 2016 [14]. We retested our lead chronotype variants while adjusting for all 15 previously described variants. LD r^2^ was calculated between our lead variant and those of 23andMe if they were within 500kb of one another by using the 120,286 unrelated white British individuals in UK Biobank. Significance is lost for variants rs516134, rs11162296, rs75804782, rs10157197, rs12140153 and rs76899638 and so we consider these to belong to the same loci as previously reported variants. Our remaining variants are still genome-wide significant.
(XLSX)

S3 Table List of 22 loci, including 15 previously described by 23andMe and 7 in this study. Where a locus is shared between studies, meta-analysis *P*-values were compared between the lead variants and the one with lowest *P*-value selected.
(XLSX)

S4 Table MAGENTA pathways reaching nominal GSEA *P*-value (95% cutoff) of 0.05 or smaller, ordered by GSEA *P*-value (95% cutoff). (XLSX)

S5 Table LD Score genetic trait correlations (rG) and *P*-values. Correlations with *P*-values < 4.95E-4 (0.05/101) are highlighted green. Those with *P*-values < 4.95E-3 are highlighted yellow.
(XLSX)

S6 Table Summary of the 76 body mass index (BMI) and 65 type 2 diabetes (T2D) SNPs used in the Mendelian Randomisation analyses. GIANT (BMI) and DIAGRAM (T2D) betas were used as weights in the genetic risk scores. UK Biobank betas (SEs) and *P*-values are reported for inverse-normalised BMI and Chronotype; log-odds ratios (SEs) and *P*-values are reported for T2D.
(XLSX)

S7 Table Results of Mendelian Randomisation analyses performed in the UK Biobank dataset. External betas (log-ORs) were used as weights to generate the genetic risk scores used in IVreg2. SNP-phenotype associations were generated in STATA using 120,286 unrelated white British individuals.
(XLSX)

S8 Table Association statistics of the 16 chronotype variants with BMI and type 2 diabetes in the UK Biobank. Type 2 diabetes ORs and SEs were generated in a smaller subset of unrelated individuals as compared to the *P*-values, owing to limitations of Linear Mixed Models method.
(XLSX)

S9 Table Output from the Broad Institute's HaploReg tool (http://www.broadinstitute.org/mammals/haploreg/haploreg.php) identifying missense coding variants within LD r^2^ ≥ 0.8 of four of the 22 lead chronotype variants. Only missense, nonsense, non-frameshift and frameshift variants are given in this table. No coding variants were found for sleep duration.
(XLSX)

S1 Fig U-shaped association between sleep duration and both self-report BMI and type 2 diabetes prevalence. Average self-report BMI (left) and type 2 diabetes prevalence (right) over each of the sleep duration categories, calculated using the full UK Biobank cohort of 502,665 individuals. Error bars indicate standard error. Average BMI or type 2 diabetes prevalence values with standard errors exceeding the plot limits were omitted.
(TIFF)
