# Supplementary figures and images for: Genome-Wide Association Analyses in 128,266 Individuals Identifies New Morningness and Sleep Duration Loci
Source: PLoS Genet. 2016 Aug 5;12(8):e1006125. doi: 10.1371/journal.pgen.1006125 (PMC4975467; doi:10.1371/journal.pgen.1006125)

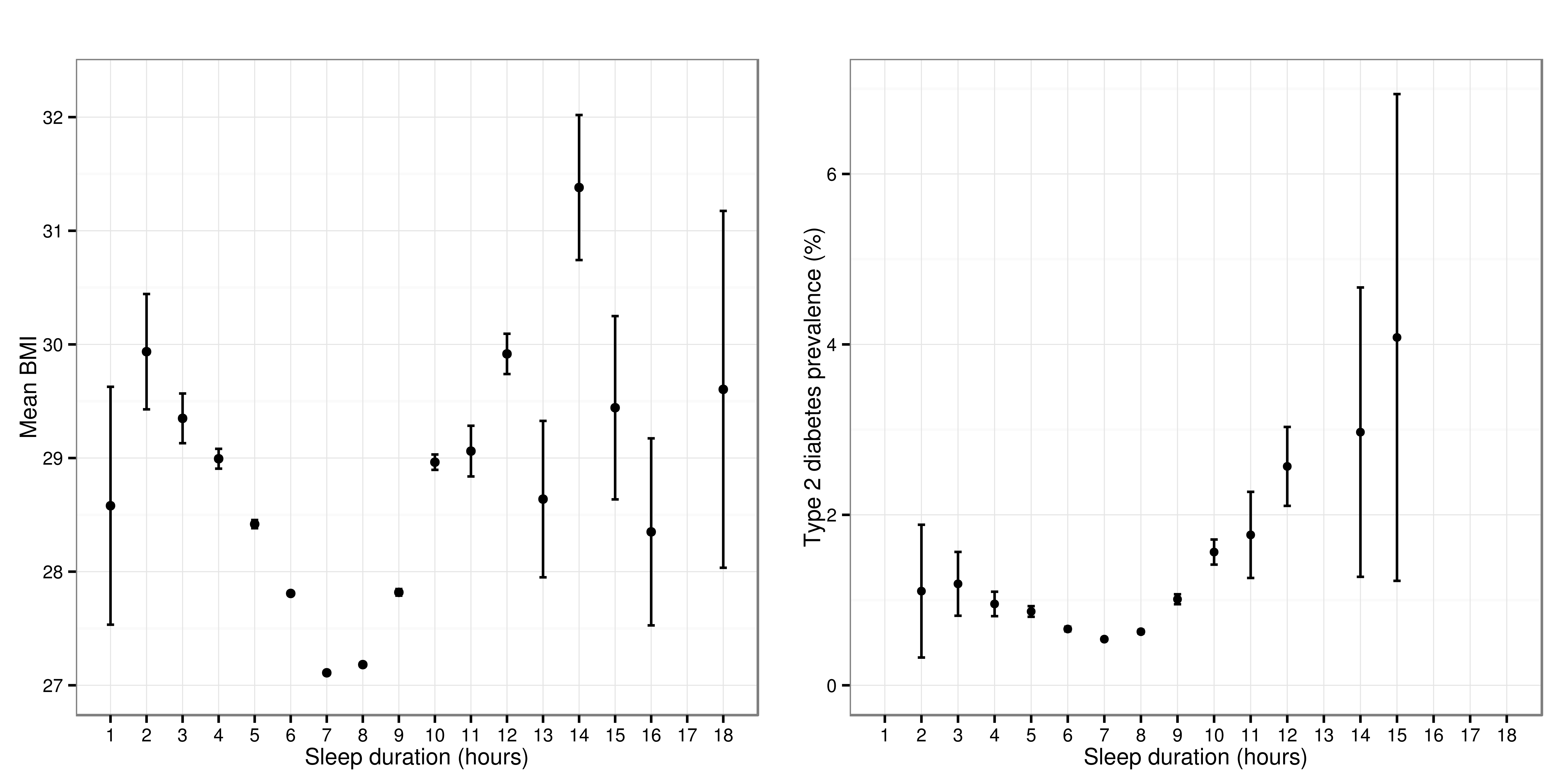

Supplement: S1 Fig — Average self-report BMI (left) and type 2 diabetes prevalence (right) over each of the sleep duration categories, calculated using the full UK Biobank cohort of 502,665 individuals. Error bars indicate standard error. Average BMI or type 2 diabetes prevalence values with standard errors exceeding the plot limits were omitted. (TIFF) [file pgen.1006125.s010.tiff]
